# Survival Outcomes With Cadonilimab Versus PD‐(L)1 Therapy in Front‐Line Gastric Cancer: A Network Meta‐Analysis

**DOI:** 10.1002/cnr2.70672

**Published:** 2026-09-15

**Authors:** Zi‐Kun Yu, Jia‐Hong Yi, Ju Xue, Yu‐Chen Huang, Yue Zhao, Chang Jiang, Chen‐Xi Yin, Biao Xia, Yi‐Xin Zhou, Wen‐Zhuo He, Liang‐Ping Xia

**Affiliations:** ^1^ VIP Region Sun Yat‐Sen University Cancer Center Guangzhou Guangdong China; ^2^ State Key Laboratory of Oncology in South China; Collaborative Innovation Center for Cancer Medicine Sun Yat‐Sen University Cancer Center Guangzhou Guangdong China; ^3^ First Clinical College Anhui Medical University Hefei China; ^4^ Department of Radiation Oncology Sun Yat‐Sen University Cancer Center Guangzhou Guangdong China

**Keywords:** cadonilimab, front‐line treatment, GC/GEJ, network meta‐analysis, PD‐(L)1 therapy, PD‐L1 expression

## Abstract

**Background:**

The FDA Oncologic Drugs Advisory Committee (ODAC) highlighted that the risk–benefit profile for patients with low PD‐L1 expression remains unclear in front‐line treatment of HER2‐negative gastric and gastroesophageal junction (GC/GEJ) cancer with PD‐(L)1 therapy. The first PD‐1/CTLA‐4 bispecific antibody (BsAb), cadonilimab, has been approved, but it is unknown if it can extend the PD‐(L)1 regimen to lower PD‐L1 patients.

**Aims:**

To indirectly compare the survival outcomes of cadonilimab versus PD‐(L)1 therapy in first‐line HER2‐negative gastric and gastroesophageal junction cancer, with particular focus on patients with low PD‐L1 expression, using a network meta‐analysis.

**Methods:**

Randomized controlled trials with ICIs as first‐line treatment were searched in public databases and FDA ODAC up to December 31, 2024. A network meta‐analysis was conducted to evaluate the efficacy and safety of approved ICI‐based treatments to refine the optimal approach for front‐line GC/GEJ.

**Results:**

A total of 7127 patients from eight Phase III trials were included. All ICIs‐based therapies significantly improved overall survival (OS) compared to chemotherapy. In indirect comparisons, cadonilimab plus XELOX was associated with numerically more favorable OS (HR = 0.78, 95% CI: 0.62–0.98) compared with PD‐(L)1 inhibitors. Numerical trends toward longer survival were observed across subgroups, including patients with PD‐L1 < 10 (HR = 0.77, 95% CI: 0.58–1.01) and liver metastasis (HR = 0.67, 95% CI: 0.49–0.93), both historically considered immunotherapy‐insensitive populations. All ICIs improved PFS compared to chemotherapy. Cadonilimab was associated with numerically better PFS versus PD‐(L)1 inhibitors in indirect comparisons (HR = 0.70, 95% CI: 0.57–0.86). The safety analysis showed no significant increase in severe adverse events.

**Conclusions:**

In this indirect comparison, cadonilimab showed more favorable survival outcomes versus PD‐(L)1 inhibitors in first‐line GC/GEJ. Subgroup benefits were observed in liver metastasis, while the PD‐L1 < 10 subgroup showed numerical trends. These results highlight the importance of selecting appropriate ICIs to optimize outcomes.

**Abbreviations**
AACR: American Association for Cancer Research
ASCO: American Society of Clinical Oncology
CI: confidence interval
CPS: combined positive score
CTLA‐4: cytotoxic T‐lymphocyte antigen‐4
ESMO: European Society for Medical Oncology
GC: gastric cancer
GEJ: gastroesophageal junction cancer
HER2: human epidermal growth factor receptor 2
HR: hazard ratio
ICI: immune checkpoint inhibitor
ITT: intention‐to‐treat
OR: odds ratio
OS: overall survival
PD‐1: programmed cell death protein‐1
PD‐L1: programmed cell death ligand‐1
PFS: progression‐free survival
TAP: tumor area positivity score
Treg: regulatory T cells

## 1 Introduction

Gastric cancer and gastroesophageal junction (GC/GEJ) cancer are the fifth most common malignancies worldwide and remain the leading causes of global cancer mortality [1]. Previous studies have found that GC/GEJ cancers are endemic in several regions, especially in Eastern Asia, where these malignancies significantly contribute to the cancer burden in terms of incidence and lethality, especially in China [1, 2]. No typical symptoms can be observed in the early stages of GC or the GEJ. Consequently, GC and the GEJ are difficult to detect at an early stage and are often diagnosed at advanced stages, even with distant metastasis [3, 4, 5]. Recently, as various studies have explored optimal treatments for locally advanced and metastatic GC and GEJ [6, 7], ICIs have demonstrated significant efficacy in this setting [8]. Several Phase III clinical trials found that immunotherapy plus chemotherapy, such as cadonilimab, pembrolizumab, nivolumab, sugemalimab, tislelizumab, and sintilimab, with chemotherapy can significantly improve the survival outcomes in patients with advanced GC and GEJ [9, 10, 11, 12, 13, 14, 15, 16]. These combinations have been approved in China as first‐line treatment for HER2‐negative advanced GC or GEJ.

Recent discussions at the 2024 FDA ODAC meeting revisited the comparative efficacy of PD‐1 inhibitors in combination with chemotherapy versus chemotherapy alone across different PD‐L1 expressing populations. The meeting concluded that patients with PD‐L1 TAP/CPS ≥ 10 derive the greatest benefit from PD‐1 inhibitor, while those with PD‐L1 < 1 show no significant improvement. The clinical impact of intermediate PD‐L1 levels (1 < PD‐L1 < 10) remains uncertain, prompting ongoing debate regarding optimal treatment strategies for this subgroup [17]. Moreover, the efficacy benefit is unclear among some subgroups with PD‐(L)1 antibody plus chemotherapy, particularly in immunotherapy‐insensitive patients, such as liver metastatic patients [9, 10, 11, 12, 13, 14, 15, 16]. In the COMPASSION‐15 trial, cadonilimab, a PD‐1/CTLA‐4 bispecific antibody (BsAb), demonstrated efficacy even in patients with low PD‐L1 expression, suggesting that PD‐1/CTLA‐4 dual inhibition might offer advantages over PD‐(L)1 monotherapy in certain GC subgroups [14]. This raises the question: Can the combination of cadonilimab and chemotherapy provide a new option for certain subgroups that do not show significant benefits from PD‐(L)1 inhibitors, especially for those with low PD‐L1 expression or liver metastasis? To address this, we conducted a network meta‐analysis to indirectly compare the efficacy of PD‐1/CTLA‐4 BsAb (cadonilimab) with PD‐(L)1 antibody in the treatment of advanced GC and GEJ.

## 2 Methods

### 2.1 Registration

This study has been registered in the PROSPERO repository under the registration number CRD42025629713. Throughout the conduct of this research, we adhered strictly to the Preferred Reporting Items for Systematic reviews and Meta‐Analyses (PRISMA) guidelines.

### 2.2 Search Strategy

#### 2.2.1 Database Search

The PubMed, Web of Science, Embase, Cochrane databases were inquired from their inception to December 31, 2024, with the following terms: “gastric, gastro‐oesophageal junction, and oesophageal adenocarcinoma,” “phase 3 trial,” and “PD‐1 OR PD‐L1” and the language was limited to English.

#### 2.2.2 Conference Abstracts

Relevant abstracts/reports/materials from conferences or meetings were searched at the same time, for example, American Society of Clinical Oncology (ASCO), European Society for Medical Oncology (ESMO), American Association for Cancer Research (AACR).

#### 2.2.3 FDA ODAC Documents

Additionally, data from the FDA ODAC [17] were used to supplement the findings identified through the database searches.

Details of the full search strategy are included in the Supporting Information Methods and Results. For trials with multiple reports (e.g., conference abstracts, interim analyses, and final publications), the most complete and mature data available were used. Duplicate or overlapping reports were excluded after cross‐checking trial identifiers (NCT number, study acronym).

### 2.3 Study Selection and Data Extraction

Publications identified by the search strategy were selected for the final network meta‐analysis using the following inclusion and exclusion criteria:

#### 2.3.1 Inclusion Criteria

(1) Studies that involved ICIs with current approved indications for the treatment of GC/GEJ. (2) Phase III randomized controlled clinical trial; (3) all patients should be pathologically diagnosed with GC/GEJ and receive first‐line therapy; (4) every treatment arm should include fluorouracil and platin‐based combination chemotherapy; (5) the experimental arm should be single‐agent anti‐PD‐(L)1 or anti‐PD‐1/CTLA‐4 bispecific antibody immunotherapy along with chemotherapy; (6) studies should report the toxicity outcome and at least one survival outcome, such as progression‐free survival (PFS) or overall survival (OS) and their 95% confidence interval (95% CI).

#### 2.3.2 Exclusion Criteria

(1) Patients were pathologically diagnosed with HER2 positive GC/GEJ; (2) research was noninterventional.

### 2.4 End Point Definitions

The primary endpoint was OS, defined as the time from randomization to death from any cause. The secondary endpoint was PFS, defined as the time from randomization to the first occurrence of disease progression or any cause of death. Toxicity evaluations and comparisons were based on the incidence of Grades 3–5 treatment‐related adverse events (TRAEs) and serious adverse events (SAEs), which were graded according to the National Cancer Institute Common Terminology Criteria for Adverse Events.

### 2.5 Quality Assessment

The risk of bias assessment for each included study was conducted independently by two reviewers (Z.‐K.‐Y. and J.‐H.Y.), utilizing the Cochrane risk‐of‐bias tool for randomized trials, version 2 (Rob 2) [18]. The tool assesses the risk of biases across five domains: the randomization process, deviations from intended interventions, missing outcome data, outcome measurement, and selection of reported results. Each domain is summarized and categorized as either low risk, with some concerns, or high risk.

### 2.6 Statistical Analysis

Primary outcome measures were pooled hazard ratio (HR) for OS, PFS, and pooled odds ratio (OR) for Grades 3–5 TRAEs and SAEs in the ICIs‐based group and chemotherapy. A subgroup analysis using OS data was conducted for the following variables: age group (< 65 and ≥ 65 years old), gender, ECOG PS, PD‐L1 expression (1, 5, 10, and), the liver metastasis, and primary tumor location (GAS or GEJ). Moreover, the OS difference between cadonilimab and single target ICI agents was compared in each subpopulation.

The network meta‐analysis for comparing HR was conducted using a frequentist approach with the R package netmeta [19, 20] in R 4.0.5 (R Software, Austria). The selection between random‐effect and fixed‐effect models for all comparisons was determined based on the results of heterogeneity test, with *I* ^2^ statistics and Cochran's *Q* test being employed to assess between‐study variations. Forest plots were created to show the efficacy and toxicity of the different therapies. Treatment efficacy was ranked using the Probability‐score, which was 0% when a treatment was certain to be the worst, and 100% when a treatment was certain to be the best [21]. Subgroup estimates were derived from study‐level reported HRs and 95% CIs, not from pooled individual patient data. Therefore, all subgroup findings should be regarded as hypothesis‐generating and should not be interpreted as evidence of patient‐level effect modification. Publication bias assessment was conducted using Egger's test and depicted through the funnel plot. Inconsistency between direct and indirect evidence was planned to be assessed using the design‐by‐treatment interaction model (global) and the SIDE method (local). However, the star‐shaped network structure—in which cadonilimab is connected to PD‐(L)1 inhibitors only via the common chemotherapy comparator—contains no closed loops. Consequently, no comparison has both direct and indirect evidence, precluding formal inconsistency testing.

All included trials employed platinum plus fluoropyrimidine doublets (XELOX, FOLFOX, SOX, FP). These regimens are considered clinically equivalent as first‐line standard‐of‐care for advanced GC/GEJ, based on evidence from pivotal Phases II and III noninferiority trials [22, 23, 24, 25, 26]. A systematic review further supports the interchangeability of these backbones [27]. Consistent with the approach of Ter Veer et al. [28], who grouped different fluoropyrimidine‐containing regimens into a common drug‐class node based on their shared mechanism of action, we treated all platinum‐fluoropyrimidine doublets as a common chemotherapy node in the network meta‐analysis.

Published evidence has consistently found no significant ethnicity‐by‐treatment interaction for OS benefit of checkpoint inhibitors in this setting [11, 15, 16, 29, 30]. Therefore, we treated the geographically diverse trial populations as jointly comparable for the indirect comparisons.

### 2.7 PD‐L1 Subgroup Harmonization

All PD‐L1 subgroup analyses were based on the combined positive score (CPS) as reported in each trial. Three cutoffs—CPS ≥ 1, ≥ 5, and ≥ 10—were used, corresponding to the pre‐specified thresholds in the original trial protocols. Although the included trials employed different IHC platforms (22C3, 28‐8, SP263), published analytical concordance studies in gastric cancer have demonstrated substantial to excellent agreement in CPS across these assays, supporting their comparability in this analysis [31, 32, 33, 34, 35].

### 2.8 Patient and Public Involvement

Patients and/or the public were not involved in the design, conduct, reporting, or dissemination plans of this research. This study is based on published literature, and patients cannot be identified. Therefore, this study does not require informed consent.

## 3 Results

### 3.1 Literature Search and Study Characteristics

**FIGURE 1:**
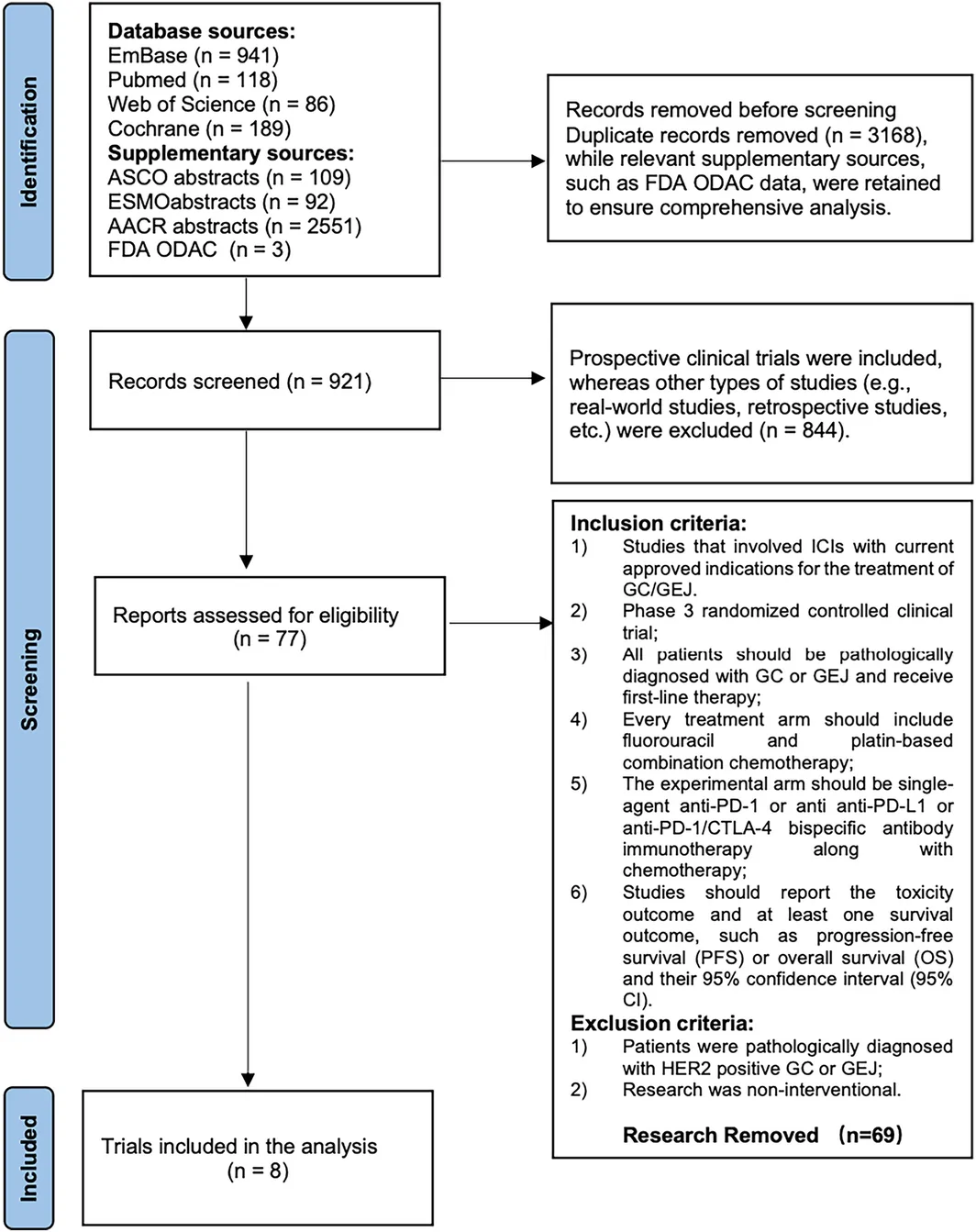
Flow chart of the research screening of the study.

The initial search resulted in 4089 potential publications from database, conference and meetings. After removing duplicates and screening the titles and abstracts, 77 prospective intervention clinical studies were identified. According to the inclusion and exclusion criteria, finally eight original studies met all the criteria and were selected for the network meta‐analysis. Totally 7127 patients from eight studies (KEYNOTE‐062 [9], ATTRACTION‐4 [10], CheckMate‐649 [16], KEYNOTE‐859 [11], GEMSTONE‐303 [12], ORIENT‐16 [13], COMPASSION‐15 [14], and RATIONALE 305 [15]) were included in the final analysis (Figure 1). The baseline characteristics of patients in the eight randomized controlled trials are presented in Table S1. Some patients with untreated, locally advanced, or metastatic HER2‐negative GC or GEJ received chemotherapy combined with platinum (oxaliplatin or cisplatin) and fluoropyrimidine alone, while others received chemotherapy and immunotherapy including anti‐PD‐(L)1 (sintilimab, nivolumab, pembrolizumab, tislelizumab, and sugemalimab) and anti‐CTLA‐4/PD‐1 bispecific antibody (cadonilimab). Among the 7127 patients, 3555 received chemotherapy and a placebo, whereas the other 3572 received chemotherapy and ICI. Details of the patient distribution and treatment plan of the eight controlled trials are shown in Tables S1 and S2.

### 3.2 Quality and Bias Assessment

Two independent reviewers evaluated the risk of bias using the Cochrane risk of bias tool (RoB 2 [18]). Since all studies were well‐designed RCTs, the risk of bias was low across all studies in general. Although some studies did not describe the methods of randomization and allocation concealment, selection and attrition bias appeared to be minimal. As all studies were analyzed based on the intention‐to‐treat (ITT) population and reported sufficient endpoints, a low risk of bias was observed with respect to incomplete outcome data and selective reporting. The results of our quality assessment are outlined in Table S3.

### 3.3 OS in ITT Population

**FIGURE 2:**
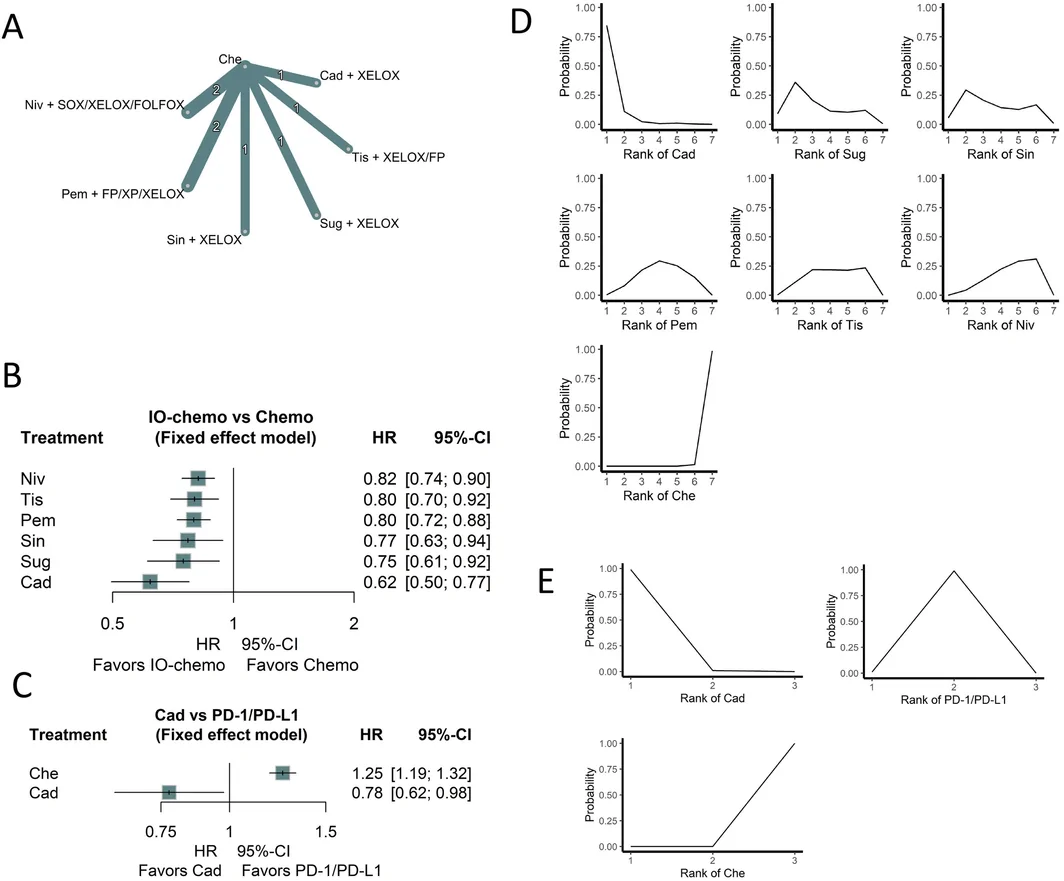
Overall survival analysis in all ITT patients. (A) Network plot of the included studies. (B) Forest plot of OS for ICI‐based therapies versus chemotherapy. (C) Forest plot comparing OS for cadonilimab plus chemotherapy versus anti‐PD‐(L)1 plus chemotherapy (reference group). (D) Treatment ranking for OS. (E) Ranking probabilities for the three treatment classes (cadonilimab plus chemotherapy, anti‐PD‐(L)1 plus chemotherapy, and chemotherapy alone). *P*‐score rankings are descriptive and should not be interpreted as definitive evidence of superiority. Cad = Cadonilimab, Che = Chemotherapy, Niv = Nivolumab, Pem = Pembrolizumab, Sin = Sintilimab, Sug = Sugemalimab, Tis = Tislelizumab.

OS was reported in all eight studies and was conducted in the analyses (Figure 2A). Six ICIs regimens in combination with chemotherapies were compared with chemotherapy plus placebo (Figure 2A). The *I* ^2^ test was 1.8%, indicating a low risk of heterogeneity within the comparison. The comparison of OS HR in the fixed effect model showed significant benefits for nivolumab (HR = 0.82, 95% CI: 0.74–0.90), tislelizumab (HR = 0.80, 95% CI: 0.70–0.92), pembrolizumab (HR = 0.80, 95% CI: 0.72–0.88), sintilimab (HR = 0.77, 95% CI: 0.63–0.94), sugemalimab (HR = 0.75, 95% CI: 0.61–0.92), and cadonilimab (HR = 0.62, 95% CI: 0.50–0.77) (Figure 2B). In the treatment effect ranking, cadonilimab demonstrated the highest efficacy (probability‐score = 0.9586), followed by sugemalimab and sintilimab as the second tier. Pembrolizumab, tislelizumab, and nivolumab ranked subsequently. All ICIs exhibited superior OS compared to chemotherapies (Figure 2D).

In indirect comparisons, cadonilimab‐based therapy was associated with numerically more favorable OS compared with anti‐PD‐(L)1 therapy (HR = 0.78, 95% CI: 0.62–0.98) (Figure 2C). Heterogeneity was low (*I* ^2^ = 0%), supporting the consistency of this estimate. The corresponding ranking analysis is shown in Figure 2E. A network league table summarizing the efficacy comparisons is presented in Table S4.

### 3.4 OS in Subpopulation

A subgroup analysis was conducted to assess OS based on different PD‐(L)1 inhibitors plus chemotherapy of intervention. The benefits of ICI plus chemotherapy varied among patients with different characteristics. Notably, consistent findings across ICI regimens indicated that the benefits for females are not clear. Patients with stomach tumors, rather than gastroesophageal junction tumors, as well as PD‐L CPS ≥ 5 patients exhibited significantly greater OS improvements with ICI combination therapy compared to chemotherapy alone. Patients under 65 years old, or with metastasis generally benefited from ICIs, except for sugemalimab in the subgroup of patients under 65 years old. Patients with distant metastasis seem to have more benefits than those with locally advanced conditions from ICIs‐based treatment. Younger patients under 65 years old and tumor location of stomach tend to be more favorable to ICIs as well. In patients with gastroesophageal junction cancer, cadonilimab, tislelizumab, and pembrolizumab each demonstrated improved OS compared with chemotherapy. However, only cadonilimab and pembrolizumab confirmed a significant OS benefit in patients with PD‐L1 CPS < 5 or < 10 (Figure S1). To identify preferred regimens across diverse patient characteristics, cadonilimab was indirectly compared with anti‐PD‐(L)1 therapies (Figure S2). Cadonilimab plus XELOX was associated with a consistent numerical trend toward improved OS across the majority of subgroups when indirectly compared with other ICI‐based therapies. The results of heterogeneity tests for subpopulations were provided in Tables S5 and S6. In the *P*‐score ranking analysis, cadonilimab plus XELOX ranked first across nearly all subgroups, with the exception of the GEJ subgroup where it shared the highest probability with sugemalimab (both approximately 40%) (Figure S3). However, these rankings, derived from indirect comparisons, should be interpreted as exploratory.

#### 3.4.1 Cadonilimab Showed a Trend Toward Improved OS in the Low PD‐L1 Expression Population (Cad vs. Anti‐PD‐(L)1)

**FIGURE 3:**
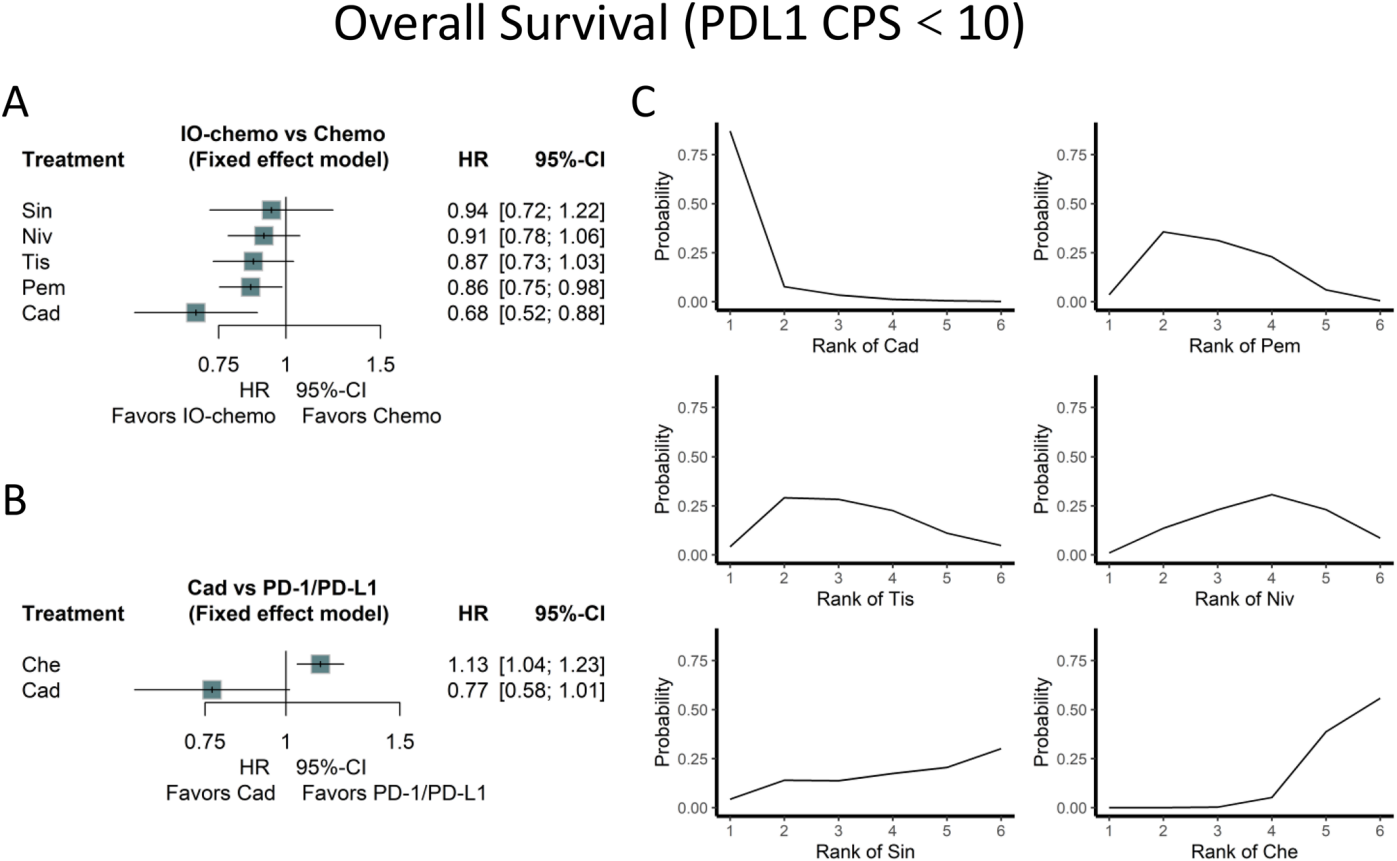
Subgroup analysis in patients with PD‐L1 expression < 10. (A) The forest plot of the OS of patients receiving ICI treatments or placebo plus chemotherapy in the fixed effect model. (B) The forest plot comparing the hazard ratio of OS of patients treated by anti‐PD‐1/PD‐L1 plus chemotherapy, cadonilimab plus chemotherapy, and chemotherapy alone (reference group: anti‐PD‐1/PD‐L1 plus chemotherapy). (C) The ranking of each treatment effect for OS. *P*‐score rankings are descriptive and should not be interpreted as definitive evidence of superiority.

The forest plot in Figure 3A shows that the HR for OS of cadonilimab combined with chemotherapy versus chemotherapy alone is 0.68 (95% CI: 0.52–0.88) in the subgroup of PD‐L1 CPS < 10 subgroup. An HR < 1 with a CI clearly indicates that cadonilimab combined with chemotherapy significantly reduces death risk versus chemotherapy alone. In contrast, the HRs of sintilimab, nivolumab, tislelizumab, and pembrolizumab combined with chemotherapy versus chemotherapy are near or above 1, indicating less pronounced benefits than cadonilimab. In the PD‐L1 < 10 subgroup, the point estimate for OS favored cadonilimab plus chemotherapy over PD‐(L)1 inhibitor‐based therapy (HR = 0.77, 95% CI: 0.58–1.01, *I* ^2^ = 0%) (Figure 3B). While the upper bound of the CI marginally crossed 1.0, the direction and magnitude of the effect were consistent with the benefits observed at the CPS < 1 and CPS < 5 cutoffs (Figure S2), suggesting a consistent trend toward improved survival across PD‐L1 low‐expression subgroups. In the ranking analysis (Figure 3C), cadonilimab occupied the most favorable position, though these probabilistic rankings should be viewed as exploratory given the statistical uncertainty in the subgroup estimates.

#### 3.4.2 Improved OS With Cadonilimab in the Liver Metastasis Subgroup (Indirect Comparison vs. Anti‐PD‐(L)1)

**FIGURE 4:**
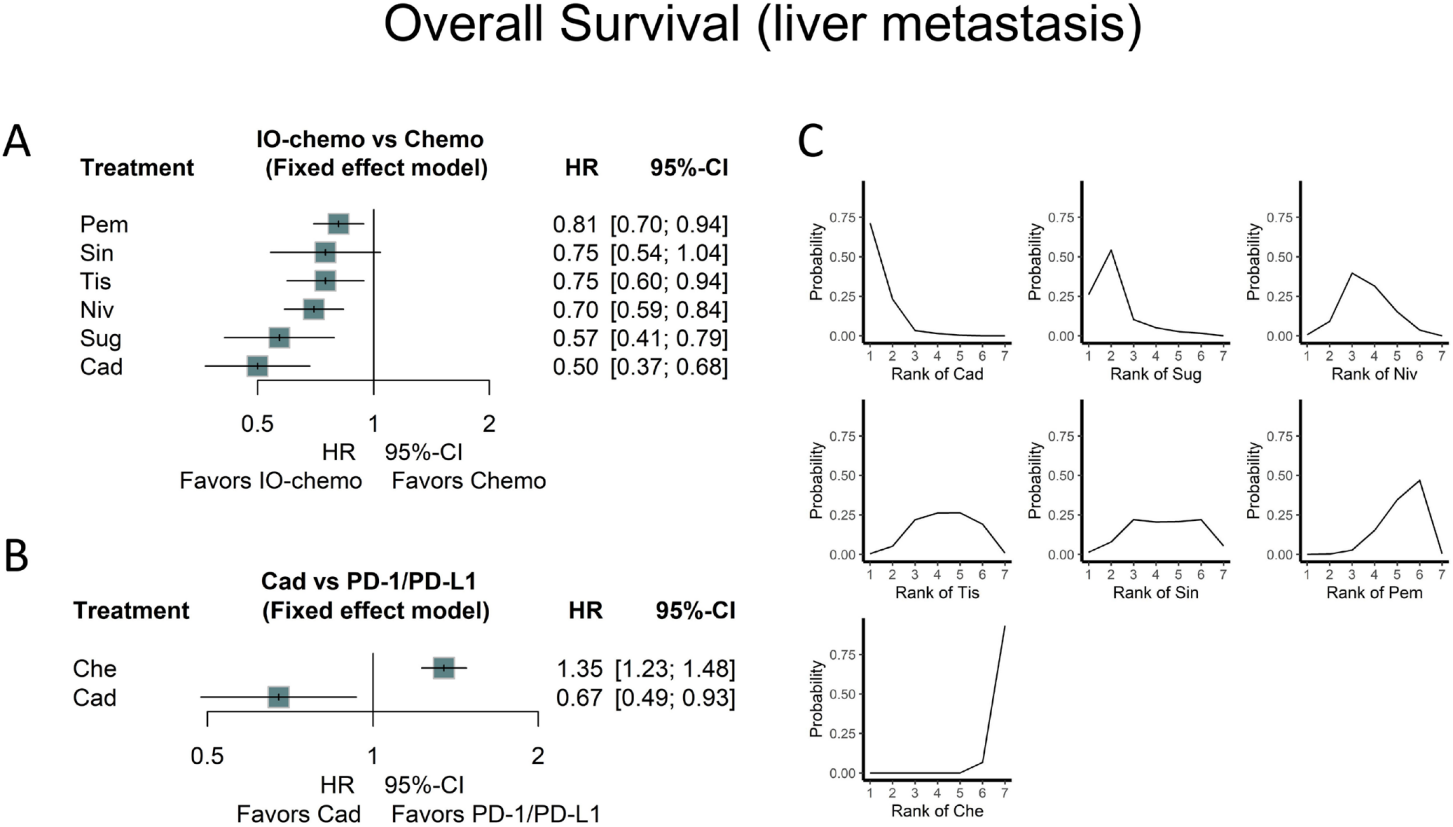
Subgroup analysis in patients with liver metastasis. (A) The forest plot of the OS of patients receiving ICI treatments or placebo plus chemotherapy in the fixed effect model. (B) The forest plot comparing the hazard ratio of OS of patients treated by anti‐PD‐1/PD‐L1 plus chemotherapy, cadonilimab plus chemotherapy, and chemotherapy alone (reference group: anti‐PD‐1/PD‐L1 plus chemotherapy). (C) The ranking of each treatment effect for OS. *P*‐score rankings are descriptive and should not be interpreted as definitive evidence of superiority.

As depicted in Figure 4, the subgroup analysis in patients with liver metastasis reveals the following: Figure 4A (OS forest plot): In the fixed‐effect model, cadonilimab plus chemotherapy shows a HR of 0.50 (95% CI: 0.37–0.68) versus chemotherapy alone. Compared with the HR estimates for other PD‐(L)1 inhibitors (pembrolizumab, sintilimab, tislelizumab, nivolumab, and sugemalimab), the point estimate for cadonilimab more distinctly favored OS in patients with liver metastasis. The probability ranking plots for OS were consistent with this observation (Figure 4C). In this indirect comparison, cadonilimab was associated with a statistically significant reduction in the hazard of death in the liver metastasis subgroup compared with anti‐PD‐(L)1 therapy (HR = 0.67, 95% CI: 0.49–0.93) (Figure 4B). Collectively, these data suggest that cadonilimab plus chemotherapy may offer improved OS compared with PD‐(L)1‐based therapy in patients with liver metastasis.

#### 3.4.3 Improved OS With Cadonilimab in the Subgroup of Male Patients With PS = 1 (Indirect Comparison vs. Anti‐PD‐(L)1)

**FIGURE 5:**
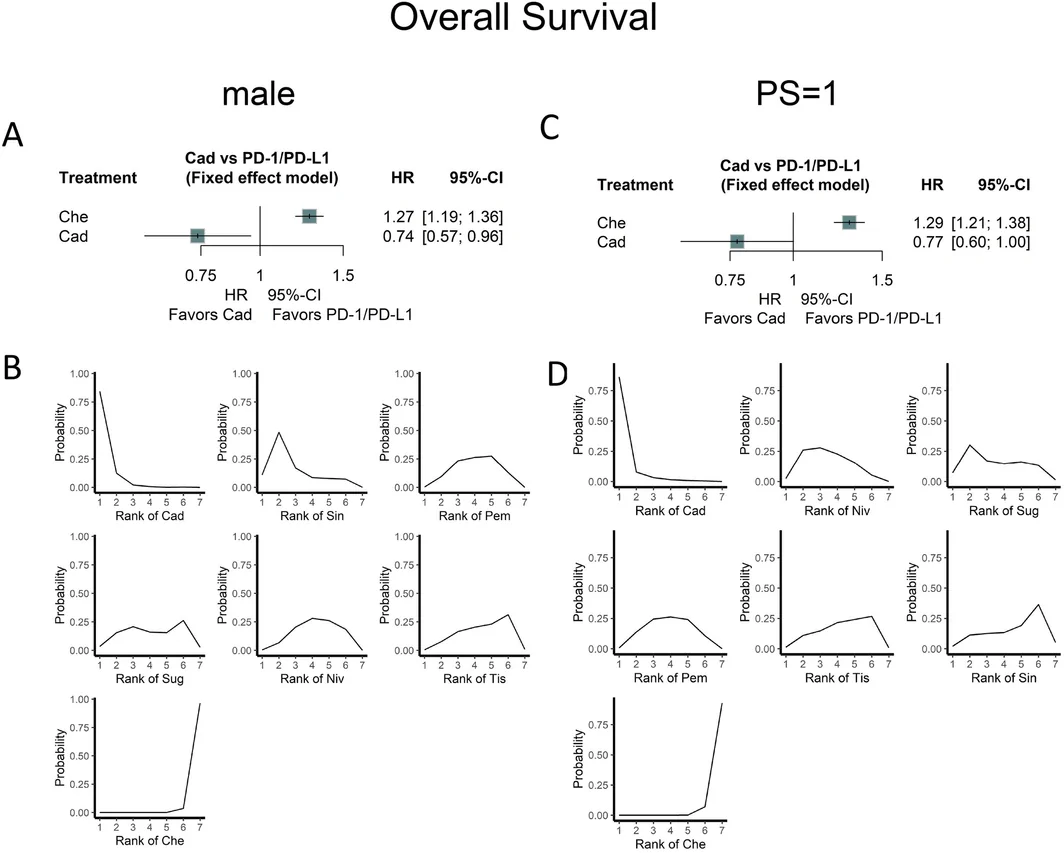
The forest plot comparing the hazard ratio of OS of subgroup patients. The forest plot comparing the hazard ratio of OS of subgroup patients with male (A) or PS = 1 (C) treated by anti‐PD‐1/PD‐L1 plus chemotherapy, cadonilimab plus chemotherapy, and chemotherapy alone (reference group: anti‐PD‐1/PD‐L1 plus chemotherapy). The ranking of each treatment effect for OS of subgroup patients with male (B) or PS = 1 (D). *P*‐score rankings are descriptive and should not be interpreted as definitive evidence of superiority.

In the subgroup of male patients (Figure 5A) and those with PS = 1 (Figure 5B), cadonilimab was associated with a statistically significant improvement in OS compared with PD‐(L)1‐based therapies, with HRs of 0.74 (95% CI: 0.57–0.96, *I* ^2^ = 0%) and 0.77 (95% CI: 0.60–1.00, *I* ^2^ = 0%), respectively. In the treatment ranking analysis, cadonilimab ranked first among all treatments in these subgroups (Figure 5C,D). Collectively, these indirect comparisons suggest that cadonilimab plus chemotherapy was associated with numerically more favorable OS compared with PD‐(L)1 inhibitors plus chemotherapy in male patients and those with PS = 1.

### 3.5 PFS

PFS results available in six ICIs regimens and eight trials was compared between ICIs plus chemotherapy and chemotherapy alone [9, 10, 11, 12, 13, 14, 15, 16]. All ICIs regimens, both single target and bispecific antibodies, showed significant benefits, pembrolizumab, tislelizumab, nivolumab, sintilimab and cadonilimab with minimal heterogeneity (*I* ^2^ = 0%) (Figure S4A). The probability analysis suggested that cadonilimab plus XELOX (probability = 0.9740) and chemotherapy without ICIs (probability = 1.0000) were ranked first and last, respectively, among the six treatment settings (Figure S4C). To further elucidate the differences among ICI treatments, cadonilimab was indirectly compared with PD‐(L)1‐based therapy (Figure S4B,D). In this analysis, cadonilimab was associated with a more favorable PFS outcome (HR = 0.70, 95% CI: 0.57–0.86; *I* ^2^ = 13.1%).

### 3.6 Toxicity of Different Treatment Regimens

Safety outcomes were assessed across all included trials [9, 10, 11, 12, 13, 14, 15]. No statistically significant differences in Grade ≥ 3 TRAEs (OR 0.83, 95% CI 0.54–1.28, *p* = 0.40) or SAEs (OR 0.89, 95% CI 0.56–1.42, *p* = 0.63) were observed between cadonilimab and PD‐(L)1 inhibitors (Figure S5A,B).

### 3.7 Publication Bias

Funnel plots for efficacy and safety endpoints exhibited symmetry around pooled effect estimates. Egger's test yielded *p* > 0.05 for OS (probability = 0.5685) and PFS (probability = 0.5666), TRAE (probability = 0.7477), and SAE (probability = 0.1930), indicating low risk of publication bias (Figure S6).

### 3.8 Sensitivity Analysis

Leave‐one‐out sensitivity analysis of OS and PFS, in which individual studies were sequentially excluded from the primary network, showed no qualitative change in the direction or statistical significance of the treatment effects, confirming the robustness of the primary findings (Figures S7 and S8).

## 4 Discussion

This network meta‐analysis, based on indirect comparisons, suggests that cadonilimab plus chemotherapy was associated with numerically more favorable OS and PFS compared with PD‐(L)1 inhibitors plus chemotherapy in patients with advanced GC/GEJ. In the ITT population, cadonilimab plus XELOX was associated with more favorable survival outcomes, with HRs of 0.78 (95% CI: 0.62–0.98) for OS and 0.70 (95% CI: 0.57–0.86) for PFS compared with PD‐(L)1 inhibitors. The treatment ranking analysis ranked cadonilimab first among all evaluated regimens. No statistically significant differences in Grade ≥ 3 TRAEs or SAEs were observed between cadonilimab and PD‐(L)1 inhibitors, though safety estimates may be imprecise. Exploratory subgroup analyses showed numerical trends toward improved OS with cadonilimab in patients with low PD‐L1 expression, liver metastasis, ECOG PS of 1, and male patients; however, these subgroup findings should be interpreted with caution. Tumor cells evade immune clearance by downregulating T‐cell responses via PD‐1 and CTLA‐4, which are expressed in 44.9% and 86.6% of gastric adenocarcinomas, respectively, and are associated with poor prognosis [36]. Inhibition on both PD‐1 and CTLA4 addresses immune evasion mechanisms more effectively than single‐agent therapies. Preclinical studies have shown that blocking both PD‐1 and CTLA‐4 produces synergistic effects, improving antitumor responses across various cancers [37, 38]. As a bispecific antibody, cadonilimab targeting both PD‐1 and CTLA‐4 simultaneously with tumor‐targeted enrichment, enhances antitumor activity [39, 40]. The combination of nivolumab and ipilimumab was also seen in CheckMate 649 trial [16]. However, the combination ICIs arm was early terminated by increasing the rate of adverse events and early deaths and did not improve PFS and OS compared to chemotherapy alone in final analysis. Different from the combination strategy of dual ICIs, cadonilimab has higher efficiency in blockage of both PD‐1 and CTLA4 given the beauty of tetravalent structure. Tetra‐body design helps drugs enrichment in local tumor environment with co‐expression of PD‐1 and CTLA4. As consequence, more efficacy and less toxicity of Cadonilimab is able to transfer the response into OS in combination with chemotherapy setting. Even though no head‐to‐head study is available, this meta‐analysis provides the first evidence from indirect comparisons favoring cadonilimab over PD‐(L)1 therapy in this setting.

The PD‐L1 expression is well known as a biomarker of PD‐(L)1 inhibitors in lung cancer, but is still not clear in gastric due to the high heterogeneity of evidence. This meta‐analysis showed a similar observation to each single‐Phase III trial. All the PD‐1 or PD‐L1 regimens drive the most clinical benefits in PD‐L1 higher score, PD‐L1 ≥ 5 or PD‐L1 ≥ 10. But there were conflicting results in patients with PD‐L1 less than 5 or 10 (Figure S1). We noted that a very high proportion of PD‐L1‐expressing patients were enrolled and negative population were excluded in the PD‐(L)1 trials (Table S1). A similar observation was also noted by FDA, prompting intensive discussion of effectiveness in the low‐expression population in the ODAC meeting. Although a few PD‐1 inhibitors were approved for first‐line gastric cancer regardless of PD‐L1 expression in past years, none of the trial results well supported the use in patients with PD‐L1 lower expression. Our data offered extra evidence and suggested not PD‐1 inhibitor but bispecific cadonilimab could be considered the optimal treatment in PD‐L1 < 10 patients (Figure 3). The dual mechanism of cadonilimab may enable antitumor activity regardless of PD‐L1 expression, as seen in the Phase III COMPASSION‐16 [41] trial of cervical cancer, whereas pembrolizumab showed limited benefit in PD‐L1‐negative patients [42].

The favorable survival outcomes of cadonilimab and chemotherapy can also be seen in patients with liver metastasis OS HR 0.67 (95% CI: 0.49–0.93) in comparison with PD‐(L)1 inhibitors. Liver metastasis is an independent poor prognosis factor in gastric cancer [43] and is associated with worse survival outcomes. The traditional chemotherapy and targeted therapy have less response and activity to liver metastasis. The underlying mechanism might be the depletion of peripheral T cells and the impairment of intratumoural T‐cell diversity and function induced by liver metastases [44]. Previous research has found that liver metastasis can cause loss of dendritic cells and CD8+ T cells, as well as upregulation of immunosuppressive regulatory T (Treg) cells and macrophages [45, 46]. Cadonilimab's dual blockade of PD‐1 and CTLA‐4 may more effectively counteract the immunosuppressive tumor microenvironment observed in liver metastases. In this study, cadonilimab plus chemotherapy was associated with a statistically significant OS improvement compared with PD‐(L)1 inhibitors plus chemotherapy in patients with gastric cancer and liver metastasis, providing preliminary support for this hypothesis. Cadonilimab monotherapy also shows good anti‐tumor activity in hepatocellular carcinoma [47]. These findings suggest that dual‐target ICIs, such as cadonilimab, may be a preferable option compared with PD‐(L)1 inhibitors for patients with liver metastasis, though this result was derived from an indirect comparison and warrants further investigation.

In addition, in indirect comparisons, cadonilimab plus chemotherapy was associated with a statistically significant improvement in OS compared with PD‐(L)1 inhibitors in male patients (HR = 0.74) and those with PS = 1 (HR = 0.77). In male patients, androgen receptor (AR) signaling in CD8+ T cells suppresses the expression of effector molecules such as IFN‐γ and promotes T cell exhaustion, thereby weakening anti‐tumor immunity [35]. Furthermore, male tumors exhibit a higher proportion of Treg cell infiltration, which is dependent on CTLA‐4 signaling to maintain their immunosuppressive function [48]. Given that cadonilimab can both suppress Treg and enhance T cell function, it may confer greater therapeutic benefits in male patients. ECOG PS = 1 patients have significantly higher levels of the neutrophil‐to‐lymphocyte ratio (NLR) and the systemic immune‐inflammation index (SII) compared to patients with PS 0 [49]. This indicates that the immune microenvironment of PS = 1 patients may exhibit a higher inflammatory state, which leads to greater T‐cell exhaustion [50]. By simultaneously blocking PD‐1 and CTLA‐4, cadonilimab may more effectively reverse this T‐cell exhaustion. It is therefore plausible that the numerically more favorable outcome observed with cadonilimab in PS = 1 patients may be explained, in part, by this mechanism.

No statistically significant differences in Grade ≥ 3 TRAEs or SAEs were observed between cadonilimab and PD‐(L)1 inhibitors (Figure S5 A&B). Previous studies of ICIs combinations in gastric and liver cancer (CM649 [16], CM9DW [51], HIMALAYA [52]) have shown that the use of CTLA‐4 inhibitors ipilimumab and tremelimumab was limited by dose‐dependent toxicity. The tetravalent structure of BsAb may promote tumor‐localized accumulation via tight binding to co‐expressed targets in the tumor microenvironment, potentially reducing peripheral blood and normal tissue toxicity [39, 40, 53]. However, these comparisons were based on a limited number of trials and the CIs for key safety endpoints were wide, precluding definitive conclusions about comparative safety. A clinically meaningful difference cannot be excluded based on current evidence.

This network meta‐analysis has several limitations mainly due to its cross‐trial nature. It is not a replacement for Level 1 prospective evidence but provides supplementary data for clinical decisions and drug development. Individual patient‐level data were unavailable, limiting the identification of baseline characteristics causing heterogeneity. All subgroup analyses in this NMA are based on study‐level aggregate data, which limits the ability to control for within‐study confounding and introduces the potential for ecological bias and should not be interpreted as evidence of patient‐level effect modification. Therefore, these subgroup findings should be regarded as hypothesis‐generating and interpreted in the context of the overall consistency of the treatment effect direction across subgroups. Different detection kits across studies may lead to heterogeneity in PD‐L1 cutoff values. Additionally, comparisons between cadonilimab and PD‐(L)1 inhibitors rely entirely on indirect evidence, as no direct head‐to‐head trial is available. SUCRA‐based treatment rankings should be interpreted cautiously, as they are sensitive to network structure and do not constitute definitive evidence of superiority. Subgroup analysis had limited power, explaining why results were significant in overall but not all subgroup analyses.

## 5 Conclusion

This network meta‐analysis indicates that cadonilimab plus XELOX may provide more favorable survival outcomes compared with PD‐(L)1 inhibitors as a first‐line treatment for advanced GC/GEJ. These findings suggest a potential role for cadonilimab among ICI‐based regimens across diverse patient subgroups. For patients with high PD‐L1 expression, all ICI‐based therapies provide a clear survival benefit over chemotherapy. In patients with liver metastasis, cadonilimab may offer a survival advantage over PD‐(L)1 inhibitors. Male patients and those with PS = 1 may also benefit from cadonilimab‐based therapy. For patients with low or negative PD‐L1 expression, cadonilimab was associated with a numerical trend toward improved OS compared with PD‐(L)1 inhibitors. These findings, derived from indirect comparisons, warrant further evaluation in prospective head‐to‐head trials.

## Author Contributions

**Zi‐Kun Yu:** conceptualization, methodology, writing – original draft. **Jia‐Hong Yi:** investigation, writing – review and editing, supervision. **Ju Xue:** investigation, formal analysis, writing – review and editing. **Yu‐Chen Huang:** writing – review and editing, investigation. **Yue Zhao:** investigation, writing – review and editing. **Chang Jiang:** formal analysis, writing – review and editing. **Chen‐Xi Yin:** investigation, supervision. **Biao Xia:** investigation, formal analysis. **Yi‐Xin Zhou:** conceptualization, supervision. **Wen‐Zhuo He:** conceptualization, funding acquisition. **Liang‐Ping Xia:** conceptualization, methodology, funding acquisition.

## Funding

This work was supported by grants from the Science and Technology Program of Guangzhou, China (2024A04J9977, SL2023A04J01560), the National Natural Science Foundation of China (82102781), and Tencent Foundation (SYSU‐84000‐20220420‐0001).

## Ethics Statement

This study is entirely based on published literature and patients cannot be identified. The studies involved in our research were approved by the clinical research committee. Therefore, this study does not require ethics approval.

## Consent

The authors have nothing to report.

## Conflicts of Interest

The authors declare no conflicts of interest.

## Supporting information


**Table S1:** The baseline characteristics of the studies included in the meta‐analysis.
**Table S2:** Baseline characteristics in the ITT population and subgroup analysis of overall survival and progression‐free survival.
**Table S3:** Version 2 of the Cochrane risk‐of‐bias assessment tool for randomized trials: bias domains, signaling questions, response options, and risk‐of‐bias judgments.
**Table S4:** Network league table based on the network meta‐analysis of the efficacy comparison between ICIs.
**Table S5:** The results of heterogeneity tests for subpopulation of OS (cadonilimab vs individual PD‐(L)1 inhibitors).
**Table S6:** The results of heterogeneity tests for subpopulation of OS (cadonilimab vs pooled PD‐(L)1 inhibitor node).
**Figure S1:** The forest plot of the OS subgroups receiving ICI treatments or placebo plus chemotherapy.
**Figure S2:** Subgroup analysis of OS comparison between patients receiving PD1/PD‐L1 plus chemotherapy and cadonilimab plus chemotherapy.
**Figure S3:** The ranking of each treatment effect for OS of subgroups.
**Figure S4:** The forest plot and the ranking of effect for PFS.
**Figure S5:** The forest plot of adverse events analysis.
**Figure S6:** The funnel plot of the analysis of efficacy and safety.
**Figure S7:** Leave‐one‐out sensitivity analysis for the primary network meta‐analysis of overall survival.
**Figure S8:** Leave‐one‐out sensitivity analysis for the primary network meta‐analysis of progression free survival.

**Supporting Information: File.** PRISMA 2020 checklist.

## Data Availability

The data that support the findings of this study are available from the corresponding author upon reasonable request.
