# Supplementary material for: Survival Outcomes With Cadonilimab Versus PD‐(L)1 Therapy in Front‐Line Gastric Cancer: A Network Meta‐Analysis
Source: Cancer Rep (Hoboken). 2026 Sep 15;9(9):e70672. doi: 10.1002/cnr2.70672 (PMC13577937; doi:10.1002/cnr2.70672)
Supplement: Supplementary file 2 [file CNR2-9-e70672-s002.docx]

**Supplementary Appendix**

**Table S1.** The baseline characteristics of the studies included in the meta-analysis.

**Table S2.** Baseline characteristics in the ITT population and subgroup analysis of overall survival and progression free survival

**Table S3.** Version 2 of the Cochrane risk-of-bias assessment tool for randomized trials: bias domains, signaling questions, response options, and risk-of-bias judgments.

**Table S4.** Network league table based on the network meta-analysis of the efficacy comparison between ICIs.

**Table S5.** The results of heterogeneity tests for subpopulation of OS (cadonilimab vs individual PD-(L)1 inhibitors).

**Table S6.** The results of heterogeneity tests for subpopulation of OS (cadonilimab vs pooled PD-(L)1 inhibitor node).

**Figure S1.** The forest plot of the OS subgroups receiving ICI treatments or placebo plus chemotherapy.

**Figure S2.** Subgroup analysis of OS comparison between patients receiving PD1/PD-L1 plus chemotherapy and cadonilimab plus chemotherapy.

**Figure S3.** The ranking of each treatment effect for OS of subgroups.

**Figure S4.** The forest plot and the ranking of effect for PFS.

**Figure S5.** The forest plot of adverse events analysis.

**Figure S6.** The funnel plot of the analysis of efficacy and safety.

**Figure S7.** Leave-one-out sensitivity analysis for the primary network meta-analysis of overall survival.

**Figure S8.** Leave-one-out sensitivity analysis for the primary network meta-analysis of progression free survival.

**Supplementary Methods and Results**

| **Source** | **Search Strategy** | **Date Searched** | **Records** |
| --- | --- | --- | --- |
| **PubMed** | **gastric cancer AND phase 3 trial AND (pd-1 OR PDL1)**  ("stomach neoplasms"[MeSH Terms] OR ("stomach"[All Fields] AND "neoplasms"[All Fields]) OR "stomach neoplasms"[All Fields] OR ("gastric"[All Fields] AND "cancer"[All Fields]) OR "gastric cancer"[All Fields]) AND (("phase"[All Fields] OR "phase s"[All Fields] OR "phases"[All Fields]) AND "3"[All Fields] AND ("clinical trials as topic"[MeSH Terms] OR ("clinical"[All Fields] AND "trials"[All Fields] AND "topic"[All Fields]) OR "clinical trials as topic"[All Fields] OR "trial"[All Fields] OR "trial s"[All Fields] OR "trialed"[All Fields] OR "trialing"[All Fields] OR "trials"[All Fields])) AND ("pd-1"[All Fields] OR "PDL1"[All Fields]). | Inception to Dec 31, 2024 | n=118 |
| **Web of Science** | **All Databases**  gastric cancer (Topic) and phase 3 trial (Topic) and pd-1 OR PDL1 (Topic) and Preprint Citation Index (Exclude – Database)  (TS=(gastric cancer) AND TS=(phase 3 trial) AND TS=(pd-1 OR PDL1)) NOT (SILOID==("PPRN")) | Inception to Dec 31, 2024 | n=86 |
| **Embase** | ('gastric cancer'/exp OR 'gastric cancer' OR (gastric AND ('cancer'/exp OR cancer))) AND phase AND 3 AND trial AND ('pd1' OR 'pdl1') | Inception to Dec 31, 2024 | n=941 |
| **Cochrane Library** | gastric cancer in Title Abstract Keyword AND phase 3 trial in Title Abstract Keyword AND pd-1 OR PDL1 in Title Abstract Keyword. (Word variations have been searched) | Inception to Dec 31, 2024 | n=189 |
| **ASCO Abstracts** | gastric cancer, phase 3 trial | Inception to Dec 31, 2024 | n=109 |
| **ESMO Abstracts** | gastric cancer AND phase 3 trial and PD-1  gastric cancer AND phase 3 trial and PD-L1 | Inception to Dec 31, 2024 | n=92 |
| **AACR Abstracts** | gastric cancer AND phase 3 trial AND pd-L1  gastric cancer AND phase 3 trial AND pd-1 | Inception to Dec 31, 2024 | n=2551 |
| **FDA ODAC (Sep 26, 2024)** | Not applicable(manual review of briefing documents) | Sep 26, 2024 | n=3 |

**FDA ODAC Briefing Documents**

The briefing documents for the Oncologic Drugs Advisory Committee meeting held on September 26, 2024, were manually reviewed to supplement unpublished subgroup data relevant to this analysis. Data for the following three trials were identified and included: **Checkmate-649, KEYNOTE-859, and RATIONALE-305**. These documents were accessed through the FDA advisory committee materials archive. <https://www.fda.gov/advisory-committees/advisory-committee-calendar/september-26-2024-meeting-oncologic-drugs-advisory-committee-meeting-announcement-09262024>.

**Table S1. The baseline characteristics of the studies included in the meta-analysis.**

|  | | **KEYNOTE-062** | **ATTRACTION-04** | **CHECKMATE-649** | **KEYNOTE-859** | **GEMSTONE-303** | **ORIENT-16** | **COMPASSION-15** | **RATIONALE-305** |  |
| --- | --- | --- | --- | --- | --- | --- | --- | --- | --- | --- |
| **Published year** | | 2020 | 2022 | 2022 | 2023 | 2023 | 2023 | 2024 | 2024 |  |
|  | |  |  |  |  |  |  |  |  |  |
| **Primary Endpoint** | **OS** | √ | √ | √ | √ | √ | √ | √ | √ |  |
|  | **PFS** | √ | √ | √ |  | √ |  |  |  |  |
|  | |  |  |  |  |  |  |  |  |  |
| **Population** | | PD-L1 CPS⩾1 pts | All randomized pts | All randomized pts | All randomized pts | PD-L1 CPS⩾5 pts | All randomized pts | All randomized pts | All randomized pts |  |
| **Age** | **＜65** | —— | 49% | 61% | 61% | 57% | 64% | 55% | 65% |  |
|  | **≥65** | —— | 51% | 39% | 39% | 43% | 36% | 45% | 35% |  |
|  |  |  |  |  |  |  |  |  |  |  |
| **Primary Tumor Location** | **GEJ** | 30% | 9% | 16% | 21% | 10% | 18% | 22% | 20% |  |
|  | **GAS** | 69% | 66% | 70% | 79% | 90% | 81% | 78% | 80% |  |
|  |  |  |  |  |  |  |  |  |  |  |
| **ECOG PS** | **0** | 48% | 54% | 42% | 37% | 25% | 28% | 23% | 32% |  |
|  | **1** | 52% | 46% | 58% | 63% | 75% | 72% | 77% | 68% |  |
|  |  |  |  |  |  |  |  |  |  |  |
| **Metastastic** | **No** | 5% | —— | 4% | 4% | 7% | 8% | 24% | 99% |  |
|  | **Yes** | 95% | —— | 96% | 96% | 93% | 92% | 76% | 1% |  |
|  |  |  |  |  |  |  |  |  |  |  |
| **Liver Metastatic** | **Yes** | —— | 36% | 39% | 40% | 39% | 39% | 47% | 38% |  |
|  |  |  |  |  |  |  |  |  |  |  |
| **Cohort (Sample size)** | | Pembrolizumab + FP/ XP (257) | Nivolumab + SOX/XELOX (362) | Nivolumab + XELOX/ FOLFOX (789) | Pembrolizumab + FP/XELOX (790) | Sugemalimab + XELOX (241) | Sintilimab + XELOX (327) | Cadonilimab + XELOX (305) | Tislelizumab + XELOX/FP (501) |  |
|  |  | Placebo + FP/ XP (250) | Placebo + SOX/XELOX (362) | Placebo + XELOX /FOLFOX (792) | Placebo + FP/XELOX (789) | Placebo + XELOX (238) | Placebo + XELOX (323) | Placebo + XELOX (305) | Placebo + XELOX/FP (496) |  |
|  | |  |  |  |  |  |  |  |  |  |
| **PD-L1 expression scoring system** | | CPS | TPS | CPS | CPS | CPS | CPS | CPS | TAP/CPS |  |
|  | |  |  |  |  |  |  |  |  |  |
| **PD-L1 IHC assays** | | 22C3 | 28—8 | 28—8 | 22C3 | SP263 | 22C3 | 22C3 | SP263 |  |
|  | |  |  |  |  |  |  |  |  |  |
| **PD-L1**⩾1 | | 100% | 16% | 82% | 78% | —— | 84% | 69% | 89% |  |
|  | |  |  |  |  |  |  |  |  |  |
| **PD-L1**⩾5 | | —— | —— | 60% | 50% | 100% | 61% | 42% | 55% |  |
|  | |  |  |  |  |  |  |  |  |  |
| **PD-L1**⩾10 | | 37% | 9% | 49% | 35% | 54% | 44% | 25% | 28% |  |
| Abbreviations: CPS, Combined Positive Score; TAP, Tumor Area Positivity; TPS, Tumor Proportion Score; FOLFOX, 5-FU + oxaliplatin + leucovorin; FP, fluorouracil + cisplatin; SOX, oxaliplatin + oral S-1; OS, overall survival; PFS, progression-free survival; XELOX, capecitabine + oxaliplatin; XP, capecitabine + cisplatin; IHC, Immunohistochemistry; pts, patients | | | | | | | | | |  |
|  |  |  |  |  |  |  |  |  |  |  |

**Table S2. Baseline characteristics in the ITT population and subgroup analysis of overall survival and progression free survival**

| Research included in the analysis | HR | HR 95%CI | Sample size |
| --- | --- | --- | --- |
| ITT population of OS |  |  | **7127** |
| COMPASSION15 | 0.62 | 0.50-0.78 | 610 |
| ORIENT16 | 0.77 | 0.63-0.94 | 650 |
| RATIONALE 305 | 0.80 | 0.70-0.92 | 997 |
| GEMSTONE-303 | 0.75 | 0.61-0.92 | 479 |
| CHECKMATE-649 | 0.79 | 0.71-0.88 | 1581 |
| KEYNOTE-859 | 0.78 | 0.70-0.87 | 1579 |
| KEYNOTE-062 | 0.85 | 0.70-1.03 | 507 |
| ATTRACTION-4 | 0.90 | 0.75-1.08 | 724 |
|  |  |  |  |
| Subgroups of OS |  |  |  |
| CPS≥1 |  |  | **4858** |
| COMPASSION15 | 0.58 | 0.44-0.75 | 420 |
| ORIENT16 | 0.73 | 0.58-0.90 | 546 |
| CHECKMATE-649 | 0.76 | 0.67-0.87 | 1296 |
| KEYNOTE-859 | 0.73 | 0.65-0.83 | 1235 |
| KEYNOTE-062 | 0.85 | 0.70-1.03 | 507 |
| RATIONALE305 | 0.78 | 0.67-0.91 | 854 |
|  |  |  |  |
| CPS＜1 |  |  | **973** |
| COMPASSION15 | 0.77 | 0.51-1.18 | 140 |
| ORIENT16 | 0.84 | 0.51-1.37 | 104 |
| CHECKMATE-649 | 0.92 | 0.70-1.23 | 265 |
| KEYNOTE-859 | 0.92 | 0.73-1.17 | 344 |
| RATIONALE305 | 1.01 | 0.66-1.52 | 120 |
|  |  |  |  |
| CPS≥5 |  |  | **3393** |
| COMPASSION15 | 0.56 | 0.39-0.80 | 256 |
| ORIENT16 | 0.66 | 0.50-0.86 | 397 |
| RATIONALE305 | 0.73 | 0.60-0.89 | 523 |
| GEMSTONE-303 | 0.75 | 0.61-0.92 | 479 |
| CHECKMATE-649 | 0.70 | 0.61-0.81 | 955 |
| KEYNOTE-859 | 0.70 | 0.60-0.82 | 783 |
|  |  |  |  |
| CPS ＜5 |  |  | **2411** |
| COMPASSION15 | 0.70 | 0.51-0.95 | 304 |
| ORIENT16 | 0.90 | 0.66-1.21 | 253 |
| RATIONALE305 | 0.89 | 0.72-1.09 | 451 |
| CHECKMATE-649 | 0.94 | 0.79-1.11 | 607 |
| KEYNOTE-859 | 0.85 | 0.73-0.98 | 796 |
|  |  |  |  |
| CPS ≥10 |  |  | **1713** |
| COMPASSION15 | 0.51 | 0.32-0.79 | 153 |
| ORIENT16 | 0.56 | 0.41-0.77 | 288 |
| KEYNOTE-859 | 0.65 | 0.53-0.79 | 551 |
| KEYNOTE-062 | 0.85 | 0.62-1.17 | 182 |
| GEMSTONE-303 | 0.64 | 0.48-0.85 | 258 |
| RATIONALE305 | 0.57 | 0.43-0.76 | 281 |
|  |  |  |  |
| CPS ＜10 |  |  | **3306** |
| COMPASSION15 | 0.68 | 0.52-0.88 | 407 |
| ORIENT16 | 0.94 | 0.72-1.22 | 362 |
| KEYNOTE-859 | 0.86 | 0.75-0.98 | 1026 |
| CHECKMATE-649 | 0.91 | 0.78-1.06 | 795 |
| RATIONALE305 | 0.91 | 0.77-1.07 | 716 |
|  |  |  |  |
| GEJ |  |  | **1311** |
| COMPASSION15 | 0.59 | 0.37-0.95 | 137 |
| ORIENT16 | 0.84 | 0.52-1.36 | 120 |
| RATIONALE305 | 0.70 | 0.51-0.97 | 196 |
| CHECKMATE-649 | 0.89 | 0.68-1.16 | 260 |
| KEYNOTE-859 | 0.74 | 0.58-0.94 | 334 |
| KEYNOTE-062 | 0.96 | 0.67-1.36 | 152 |
| ATTRACTION-4 | 1.00 | 0.55-1.82 | 62 |
| GEMSTONE-303 | 0.59 | 0.29-1.20 | 50 |
|  |  |  |  |
| GAS |  |  | **5410** |
| COMPASSION15 | 0.62 | 0.49-0.79 | 473 |
| ORIENT16 | 0.72 | 0.58-0.89 | 529 |
| RATIONALE305 | 0.83 | 0.71-0.97 | 800 |
| CHECKMATE-649 | 0.75 | 0.66-0.86 | 1110 |
| KEYNOTE-859 | 0.77 | 0.68-0.87 | 1243 |
| KEYNOTE-062 | 0.81 | 0.65-1.03 | 351 |
| ATTRACTION-4 | 0.87 | 0.70-1.09 | 475 |
| GEMSTONE-303 | 0.76 | 0.61-0.94 | 429 |
|  |  |  |  |
| ＜65 years old |  |  | **4247** |
| COMPASSION15 | 0.62 | 0.47-0.82 | 334 |
| ORIENT16 | 0.73 | 0.57-0.94 | 415 |
| RATIONALE305 | 0.81 | 0.68-0.96 | 653 |
| CHECKMATE-649 | 0.80 | 0.69-0.92 | 961 |
| KEYNOTE-859 | 0.76 | 0.66-0.88 | 965 |
| KEYNOTE-062 | 0.75 | 0.59-0.97 | 291 |
| ATTRACTION-4 | 0.79 | 0.61-1.01 | 356 |
| GEMSTONE-303 | 0.80 | 0.61-1.06 | 272 |
|  |  |  |  |
| ≥65 years old |  |  | **2880** |
| COMPASSION15 | 0.61 | 0.43-0.85 | 276 |
| ORIENT16 | 0.76 | 0.55-1.07 | 235 |
| RATIONALE305 | 0.79 | 0.61-1.01 | 344 |
| CHECKMATE-649 | 0.76 | 0.64-0.91 | 620 |
| KEYNOTE-859 | 0.77 | 0.64-0.92 | 614 |
| KEYNOTE-062 | 0.96 | 0.72-1.29 | 216 |
| ATTRACTION-4 | 1.01 | 0.77-1.31 | 368 |
| GEMSTONE-303 | 0.68 | 0.50-0.93 | 207 |
|  |  |  |  |
| Female |  |  | **2060** |
| COMPASSION15 | 0.81 | 0.52-1.26 | 136 |
| ORIENT16 | 0.84 | 0.57-1.23 | 167 |
| RATIONALE305 | 0.79 | 0.61-1.02 | 305 |
| CHECKMATE-649 | 0.85 | 0.70-1.03 | 481 |
| KEYNOTE-859 | 0.76 | 0.63-0.92 | 508 |
| KEYNOTE-062 | 0.89 | 0.62-1.29 | 133 |
| ATTRACTION-4 | 0.99 | 0.70-1.39 | 201 |
| GEMSTONE-303 | 0.65 | 0.44-0.98 | 129 |
|  |  |  |  |
| Male |  |  | **5067** |
| COMPASSION15 | 0.58 | 0.45-0.74 | 474 |
| ORIENT16 | 0.71 | 0.56-0.90 | 483 |
| RATIONALE305 | 0.81 | 0.68-0.96 | 692 |
| CHECKMATE-649 | 0.76 | 0.66-0.86 | 1100 |
| KEYNOTE-859 | 0.77 | 0.67-0.88 | 1071 |
| KEYNOTE-062 | 0.84 | 0.67-1.05 | 374 |
| ATTRACTION-4 | 0.87 | 0.70-1.07 | 523 |
| GEMSTONE-303 | 0.79 | 0.62-1.00 | 350 |
|  |  |  |  |
| ECOG PS = 0 |  |  | **2631** |
| COMPASSION15 | 0.69 | 0.44-1.09 | 142 |
| ORIENT16 | 0.55 | 0.37-0.83 | 180 |
| RATIONALE305 | 0.79 | 0.62-1.01 | 323 |
| CHECKMATE-649 | 0.81 | 0.69-0.97 | 664 |
| KEYNOTE-859 | 0.73 | 0.60-0.88 | 582 |
| KEYNOTE-062 | 0.90 | 0.67-1.21 | 233 |
| ATTRACTION-4 | 0.99 | 0.77-1.28 | 389 |
| GEMSTONE-303 | 0.70 | 0.45-1.09 | 118 |
|  |  |  |  |
| ECOG PS = 1 |  |  | **4491** |
| COMPASSION15 | 0.60 | 0.47-0.77 | 468 |
| ORIENT16 | 0.82 | 0.65-1.04 | 470 |
| RATIONALE305 | 0.8 | 0.68-0.96 | 674 |
| CHECKMATE-649 | 0.74 | 0.65-0.85 | 913 |
| KEYNOTE-859 | 0.77 | 0.68-0.89 | 997 |
| KEYNOTE-062 | 0.79 | 0.61-1.01 | 273 |
| ATTRACTION-4 | 0.81 | 0.63-1.05 | 335 |
| GEMSTONE-303 | 0.76 | 0.60-0.96 | 361 |
|  |  |  |  |
| Without distant metastasis |  |  | **93** |
| COMPASSION15 | 0.43 | 0.16-1.17 | 30 |
| ORIENT16 | 0.61 | 0.28-1.33 | 51 |
| RATIONALE305 | 0.21 | 0.04-1.11 | 12 |
|  |  |  |  |
| With distant metastasis |  |  | **3682** |
| COMPASSION15 | 0.63 | 0.51-0.79 | 580 |
| ORIENT16 | 0.75 | 0.61-0.92 | 598 |
| RATIONALE305 | 0.81 | 0.70-0.94 | 984 |
| KEYNOTE-859 | 0.77 | 0.69-0.86 | 1520 |
|  |  |  |  |
| Without Liver Metastatic |  |  | **3961** |
| COMPASSION15 | 0.75 | 0.56-1.01 | 323 |
| ORIENT16 | 0.73 | 0.57-0.94 | 395 |
| RATIONALE305 | 0.83 | 0.70-1.00 | 619 |
| CHECKMATE-649 | 0.85 | 0.74-0.98 | 918 |
| KEYNOTE-859 | 0.73 | 0.63-0.84 | 953 |
| ATTRACTION-4 | 1.00 | 0.79-1.25 | 460 |
| GEMSTONE-303 | 0.85 | 0.65-1.12 | 293 |
|  |  |  |  |
| With Liver Metastatic |  |  | **2609** |
| COMPASSION15 | 0.50 | 0.37-0.69 | 287 |
| ORIENT16 | 0.75 | 0.54-1.04 | 255 |
| RATIONALE305 | 0.75 | 0.60-0.95 | 378 |
| CHECKMATE-649 | 0.70 | 0.59-0.84 | 614 |
| KEYNOTE-859 | 0.83 | 0.70-0.99 | 625 |
| ATTRACTION-4 | 0.76 | 0.56-1.01 | 264 |
| GEMSTONE-303 | 0.57 | 0.41-0.79 | 186 |
|  |  |  |  |
| Research included in the analysis | **HR** | **HR 95%CI** | **Sample size** |
| ITT population of PFS |  |  | **7127** |
| COMPASSION15 | 0.53 | 0.44-0.65 | 610 |
| ORIENT16 | 0.64 | 0.52-0.77 | 650 |
| RATIONALE 305 | 0.78 | 0.67-0.90 | 997 |
| GEMSTONE-303 | 0.66 | 0.54-0.81 | 479 |
| CHECKMATE-649 | 0.79 | 0.70-0.89 | 1581 |
| KEYNOTE-859 | 0.76 | 0.67-0.85 | 1579 |
| KEYNOTE-062 | 0.84 | 0.70-1.02 | 507 |
| ATTRACTION-4 | 0.68 | 0.51-0.90 | 724 |

GEJ: Gastro-esophageal junction cancer; GAS: Gastric cancer; CPS: PD-L1 combined positive score.

**Table S3. Version 2 of the Cochrane risk-of-bias assessment tool for randomized trials: bias domains, signaling questions, response options, and risk-of-bias judgments**

|  | COMPASSION15 | | | ORIENT16 | | | RATIONALE305 | | | GEMSTONE-303 | | |
| --- | --- | --- | --- | --- | --- | --- | --- | --- | --- | --- | --- | --- |
| Bias domain and signaling question* | Response options | | | | | | | | | | | |
|  | Lower risk of bias | Higher risk of bias | Other | Lower risk of bias | Higher risk of bias | Other | Lower risk of bias | Higher risk of bias | Other | Lower risk of bias | Higher risk of bias | Other |
| **Bias arising from the randomization process** | | | | | | | | | | | | |
| 1.1 Was the allocation sequence random? | N | PN | N | N | PN | N | N | PN | N | N | PN | N |
| 1.2 Was the allocation sequence concealed until participants were enrolled and assigned to interventions? | N | PN | N | N | PN | N | N | PN | N | N | PN | N |
| 1.3 Did baseline differences between intervention groups suggest a problem with the randomization process? | N | PN | N | N | PN | N | N | PN | N | N | PN | N |
| Risk-of-bias judgment (low/high/some concerns) | Low | | | Low | | | Low | | | Low | | |
| Optional: What is the predicted direction of bias arising from the randomization process? | NA | | | NA | | | NA | | | NA | | |
| **Bias due to deviations from intended interventions** | | | | | | | | | | | | |
| 2.1 Were participants aware of their assigned intervention during the trial? | N | PN | N | N | PN | N | N | PN | N | N | PN | N |
| 2.2 Were carers and people delivering the interventions aware of participants’ assigned intervention during the trial? | NI | NI | NI | NI | NI | NI | NI | NI | NI | NI | NI | NI |
| 2.3 If Y/PY/NI to 2.1 or 2.2: Were there deviations from the intended intervention that arose because of the trial context? | PN | PN | N | PN | PN | N | PN | PN | N | PN | PN | N |
| 2.4 If Y/PY/NI to 2.3: Were these deviations likely to have affected the outcome? | NA | NA | NA | NA | NA | NA | NA | NA | NA | NA | NA | NA |
| 2.5 If Y/PY to 2.4: Were these deviations from intended intervention balanced between groups? | NA | NA | NA | NA | NA | NA | NA | NA | NA | NA | NA | NA |
| 2.6 Was an appropriate analysis used to estimate the effect of assignment to intervention? | PN | PN | N | PN | PN | N | PN | PN | N | PN | PN | N |
| 2.7 If N/PN/NI to 2.6: Was there potential for a substantial impact (on the result) of the failure to analyse participants in the group to which they were randomised? | PN | PN | N | PN | PN | N | PN | PN | N | PN | PN | N |
| Risk-of-bias judgment (low/high/some concerns) | Low | | | Low | | | Low | | | Low | | |
| Optional: What is the predicted direction of bias due to deviations from intended interventions? | NA | | | NA | | | NA | | | NA | | |
| **Bias due to missing outcome data** | | | | | | | | | | | | |
| 3.1 Were data for this outcome available for all, or nearly all, participants randomised? | PN | N | N | PN | N | N | PN | N | N | PN | N | N |
| 3.2 If N/PN/NI to 3.1: Is there evidence that the result was not biased by missing outcome data? | NI | NI | NI | NI | NI | NI | NI | NI | NI | NI | NI | NI |
| 3.3 If N/PN to 3.2: Could missingness in the outcome depend on its true value? | NA | NA | NA | NA | NA | NA | NA | NA | NA | NA | NA | NA |
| 3.4 If Y/PY/NI to 3.3: Is it likely that missingness in the outcome depended on its true value? | NA | NA | NA | NA | NA | NA | NA | NA | NA | NA | NA | NA |
| Risk-of-bias judgment (low/high/some concerns) | Low | | | Low | | | Low | | | Low | | |
| Optional: What is the predicted direction of bias due to missing outcome data? | NA | | | NA | | | NA | | | NA | | |
| **Bias in measurement of the outcome** | | | | | | | | | | | | |
| 4.1 Was the method of measuring the outcome inappropriate? | N | N | N | N | N | N | N | N | N | N | N | N |
| 4.2 Could measurement or ascertainment of the outcome have differed between intervention groups? | PN | PN | N | PN | PN | N | PN | PN | N | PN | PN | N |
| 4.3 If N/PN/NI to 4.1 and 4.2: Were outcome assessors aware of the intervention received by study participants? | N | N | N | N | N | N | N | N | N | N | N | N |
| 4.4 If Y/PY/NI to 4.3: Could assessment of the outcome have been influenced by knowledge of intervention received? | NA | NA | NA | NA | NA | NA | NA | NA | NA | NA | NA | NA |
| 4.5 If Y/PY/NI to 4.4: Is it likely that assessment of the outcome was influenced by knowledge of intervention received? | NA | NA | NA | NA | NA | NA | NA | NA | NA | NA | NA | NA |
| Risk-of-bias judgment (low/high/some concerns) | Low | | | Low | | | Low | | | Low | | |
| Optional: What is the predicted direction of bias in measurement of the outcome? | NA | | | NA | | | NA | | | NA | | |
| **Bias in selection of the reported result** | | | | | | | | | | | | |
| 5.1 Were the data that produced this result analysed in accordance with a prespecified analysis plan that was finalised before unblinded outcome data were available for analysis? | PN | N | N | PN | N | N | PN | N | N | PN | N | N |
| Is the numerical result being assessed likely to have been selected, on the basis of the results, from: | | | | | | | | | | | | |
| 5.2 ... multiple eligible outcome measurements (eg, scales, definitions, time points) within the outcome domain? | PN | N | N | PN | N | N | PN | N | N | PN | N | N |
| 5.3 ... multiple eligible analyses of the data? | PN | N | N | PN | N | N | PN | N | N | PN | N | N |
| Risk-of-bias judgment (low/high/some concerns) | Low | | | Low | | | Low | | | Low | | |
| Optional: What is the predicted direction bias due to selection of the reported results? | NA | | | NA | | | NA | | | NA | | |
| **Overall bias** | | | | | | | | | | | | |
| Risk-of-bias judgment (low/high/some concerns) | Low | | | Low | | | Low | | | Low | | |
| Optional: What is the overall predicted direction of bias for this outcome? | NA | | | NA | | | NA | | | NA | | |
| Y=yes; PY=probably yes; PN=probably no; N=no; NA=not applicable; NI=no information.  *Signalling questions for bias due to deviations from intended interventions relate to the effect of assignment to intervention. | | | | | | | | | | | | |

**Table S3. Version 2 of the Cochrane risk-of-bias assessment tool for randomized trials: bias domains, signaling questions, response options, and risk-of-bias judgments (continued)**

|  | CheckMate-649 | | | KEYNOTE-859 | | | KEYNOTE-062 | | | ATTRACTION-4 | | |
| --- | --- | --- | --- | --- | --- | --- | --- | --- | --- | --- | --- | --- |
| Bias domain and signaling question* | Response options | | | | | | | | | | | |
|  | Lower risk of bias | Higher risk of bias | Other | Lower risk of bias | Higher risk of bias | Other | Lower risk of bias | Higher risk of bias | Other | Lower risk of bias | Higher risk of bias | Other |
| **Bias arising from the randomization process** | | | | | | | | | | | | |
| 1.1 Was the allocation sequence random? | N | PN | N | N | PN | N | N | PN | N | N | PN | N |
| 1.2 Was the allocation sequence concealed until participants were enrolled and assigned to interventions? | N | PN | N | N | PN | N | N | PN | N | N | PN | N |
| 1.3 Did baseline differences between intervention groups suggest a problem with the randomization process? | N | PN | N | N | PN | N | N | PN | N | N | PN | N |
| Risk-of-bias judgment (low/high/some concerns) | Low | | | Low | | | Low | | | Low | | |
| Optional: What is the predicted direction of bias arising from the randomization process? | NA | | | NA | | | NA | | | NA | | |
| **Bias due to deviations from intended interventions** | | | | | | | | | | | | |
| 2.1 Were participants aware of their assigned intervention during the trial? | N | PN | N | N | PN | N | N | PN | N | N | PN | N |
| 2.2 Were carers and people delivering the interventions aware of participants’ assigned intervention during the trial? | NI | NI | NI | NI | NI | NI | NI | NI | NI | NI | NI | NI |
| 2.3 If Y/PY/NI to 2.1 or 2.2: Were there deviations from the intended intervention that arose because of the trial context? | PN | PN | N | PN | PN | N | PN | PN | N | PN | PN | N |
| 2.4 If Y/PY/NI to 2.3: Were these deviations likely to have affected the outcome? | NA | NA | NA | NA | NA | NA | NA | NA | NA | NA | NA | NA |
| 2.5 If Y/PY to 2.4: Were these deviations from intended intervention balanced between groups? | NA | NA | NA | NA | NA | NA | NA | NA | NA | NA | NA | NA |
| 2.6 Was an appropriate analysis used to estimate the effect of assignment to intervention? | PN | PN | N | PN | PN | N | PN | PN | N | PN | PN | N |
| 2.7 If N/PN/NI to 2.6: Was there potential for a substantial impact (on the result) of the failure to analyze participants in the group to which they were randomized? | PN | PN | N | PN | PN | N | PN | PN | N | PN | PN | N |
| Risk-of-bias judgment (low/high/some concerns) | Low | | | Low | | | Low | | | Low | | |
| Optional: What is the predicted direction of bias due to deviations from intended interventions? | NA | | | NA | | | NA | | | NA | | |
| **Bias due to missing outcome data** | | | | | | | | | | | | |
| 3.1 Were data for this outcome available for all, or nearly all, participants randomized? | PN | N | N | PN | N | N | PN | N | N | PN | N | N |
| 3.2 If N/PN/NI to 3.1: Is there evidence that the result was not biased by missing outcome data? | NI | NI | NI | NI | NI | NI | NI | NI | NI | NI | NI | NI |
| 3.3 If N/PN to 3.2: Could missingness in the outcome depend on its true value? | NA | NA | NA | NA | NA | NA | NA | NA | NA | NA | NA | NA |
| 3.4 If Y/PY/NI to 3.3: Is it likely that missingness in the outcome depended on its true value? | NA | NA | NA | NA | NA | NA | NA | NA | NA | NA | NA | NA |
| Risk-of-bias judgment (low/high/some concerns) | Low | | | Low | | | Low | | | Low | | |
| Optional: What is the predicted direction of bias due to missing outcome data? | NA | | | NA | | | NA | | | NA | | |
| **Bias in measurement of the outcome** | | | | | | | | | | | | |
| 4.1 Was the method of measuring the outcome inappropriate? | N | N | N | N | N | N | N | N | N | N | N | N |
| 4.2 Could measurement or ascertainment of the outcome have differed between intervention groups? | PN | PN | N | PN | PN | N | PN | PN | N | PN | PN | N |
| 4.3 If N/PN/NI to 4.1 and 4.2: Were outcome assessors aware of the intervention received by study participants? | N | N | N | N | N | N | N | N | N | N | N | N |
| 4.4 If Y/PY/NI to 4.3: Could assessment of the outcome have been influenced by knowledge of intervention received? | NA | NA | NA | NA | NA | NA | NA | NA | NA | NA | NA | NA |
| 4.5 If Y/PY/NI to 4.4: Is it likely that assessment of the outcome was influenced by knowledge of intervention received? | NA | NA | NA | NA | NA | NA | NA | NA | NA | NA | NA | NA |
| Risk-of-bias judgment (low/high/some concerns) | Low | | | Low | | | Low | | | Low | | |
| Optional: What is the predicted direction of bias in measurement of the outcome? | NA | | | NA | | | NA | | | NA | | |
| **Bias in selection of the reported result** | | | | | | | | | | | | |
| 5.1 Were the data that produced this result analyzed in accordance with a prespecified analysis plan that was finalized before unblinded outcome data were available for analysis? | PN | N | N | PN | N | N | PN | N | N | PN | N | N |
| Is the numerical result being assessed likely to have been selected, on the basis of the results, from: | | | | | | | | | | | | |
| 5.2 ... multiple eligible outcome measurements (eg, scales, definitions, time points) within the outcome domain? | PN | N | N | PN | N | N | PN | N | N | PN | N | N |
| 5.3 ... multiple eligible analyses of the data? | PN | N | N | PN | N | N | PN | N | N | PN | N | N |
| Risk-of-bias judgment (low/high/some concerns) | Low | | | Low | | | Low | | | Low | | |
| Optional: What is the predicted direction bias due to selection of the reported results? | NA | | | NA | | | NA | | | NA | | |
| **Overall bias** | | | | | | | | | | | | |
| Risk-of-bias judgment (low/high/some concerns) | Low | | | Low | | | Low | | | Low | | |
| Optional: What is the overall predicted direction of bias for this outcome? | NA | | | NA | | | NA | | | NA | | |
| Y=yes; PY=probably yes; PN=probably no; N=no; NA=not applicable; NI=no information.  *Signaling questions for bias due to deviations from intended interventions relate to the effect of assignment to intervention. | | | | | | | | | | | | |

**Table S4.** **Network league table based on the network meta-analysis of the efficacy comparison between ICIs.**

**Table S4.** (A) A network league table based on the network meta-analysis of OS comparison between ICIs. (B) A network league table based on the network meta-analysis of OS comparison between PD-1/PD-L1 plus chemotherapy and cadonilimab plus chemotherapy

**Table S5. The results of heterogeneity tests for subpopulation of OS (cadonilimab vs individual PD-(L)1 inhibitors).**

| **Subgroups** | **Cochran's Q test (p-value)*** | **df** | **I^2** |
| --- | --- | --- | --- |
| **Gender** |  |  |  |
| Female | 1.14 (0.5656) | 2 | 0% [0.0%; 89.6%] |
| Male | 1.54 (0.4621) | 2 | 0% [0.0%; 89.6%] |
| **Age** |  |  |  |
| ≥65 | 4.64 (0.0981) | 2 | 56.9% [0.0%; 87.7%] |
| ＜65 | 0.02 (0.9923) | 2 | 0% [0.0%; 89.6%] |
| **PD-L1** |  |  |  |
| ≥1 | 1.37(0.2413) | 1 | 27.2% |
| ＜1 | 0 | 0 | / |
| ≥5 | 0 | 0 | / |
| ＜5 | 0 | 0 | / |
| ≥10 | 1.97 (0.1609) | 1 | 49.1% |
| ＜10 | 0 | 0 | / |
| **Primary Tumor location** |  |  |  |
| GEJ | 1.54 (0.4632) | 2 | 0% [0.0%; 89.6%] |
| GAS | 1.42 (0.4926) | 2 | 0% [0.0%; 89.6%] |
| **ECOG PS** |  |  |  |
| 0 | 3.01 (0.2219) | 2 | 33.6% [0.0%; 78.1%] |
| 1 | 0.41 (0.8155) | 2 | 0% [0.0%; 89.6%] |
| **Metastatic** |  |  |  |
| No | 0 | 0 | / |
| Yes | 0 | 0 | / |
| **Liver Metastatic** |  |  |  |
| No | 5.19 (0.0227) | 1 | 80.7% [17.6%; 95.5%] |
| Yes | 0.25 (0.6136) | 1 | 0% |

^*^: Cochran’s Q statistic: $Q=\sum_{i=1}^{k} w_{i}{(\mathrm{TE}_{i}-\bar{\mathrm{TE}})}^{2}, df=k-1$; $I^{2}=max(0,\frac{Q-df}{Q})\times$100% (k = number of studies; $\mathrm{TE}_{i}$ = effect size of individual study; $\bar{\mathrm{TE}}$ = pooled effect size; $w_{i}$ = study weight).

/: No I^2 statistic was presented because each pairwise comparison included only one study. The degrees of freedom (df = k-1) were zero, so heterogeneity could not be quantified.

**Table S6. The results of heterogeneity tests for subpopulation of OS (cadonilimab vs pooled PD-(L)1 inhibitor node).**

| **Subgroups** | **Cochran's Q test (p-value)*** | **df** | **I^2** |
| --- | --- | --- | --- |
| **Gender** |  |  |  |
| Female | 3.47 (0.7483) | 6 | 0% [0.0%; 70.8%] |
| Male | 2.38 (0.8821) | 6 | 0% [0.0%; 70.8%] |
| **Age** |  |  |  |
| ≥65 | 6.11 (0.4112) | 6 | 1.8% [0.0%; 71.3%] |
| ＜65 | 0.84 (0.9911) | 6 | 0% [0.0%; 70.8%] |
| **PD-L1** |  |  |  |
| ≥1 | 1.88 (0.7572) | 4 | 0% [0.0%; 79.2%] |
| ＜1 | 0.34 (0.9516) | 3 | 0% [0.0%; 84.7%] |
| ≥5 | 0.70 (0.9517) | 4 | 0% [0.0%; 79.2%] |
| ＜5 | 0.78 (0.8551) | 3 | 0% [0.0%; 84.7%] |
| ≥10 | 3.59 (0.6102) | 5 | 0% [0.0%; 74.6%] |
| ＜10 | 0.54 (0.9097) | 3 | 0% [0.0%; 84.7%] |
| **Primary Tumor location** |  |  |  |
| GEJ | 3.96 (0.6828) | 6 | 0% [0.0%; 70.8%] |
| GAS | 2.62 (0.8550) | 6 | 0% [0.0%; 70.8%] |
| **ECOG PS** |  |  |  |
| 0 | 7.13 (0.2115) | 5 | 29.8% [0.0%; 71.4%] |
| 1 | 0.98 (0.9864) | 6 | 0% [0.0%; 70.8%] |
| **Metastatic** |  |  |  |
| No | 1.30 (0.2548) | 1 | 22.9% |
| Yes | 0.44 (0.8013) | 2 | 0% [0.0%; 89.6%] |
| **Liver Metastatic** |  |  |  |
| No | 6.53 (0.2582) | 5 | 23.4% [0.0%; 67.3%] |
| Yes | 4.55 (0.4737) | 5 | 0% [0.0%; 74.6%] |

^*^: Cochran’s Q statistic: $Q=\sum_{i=1}^{k} w_{i}{(\mathrm{TE}_{i}-\bar{\mathrm{TE}})}^{2}, df=k-1$; $I^{2}=max(0,\frac{Q-df}{Q})\times$100% (k = number of studies; $\mathrm{TE}_{i}$ = effect size of individual study; $\bar{\mathrm{TE}}$ = pooled effect size; $w_{i}$ = study weight).

**Figure S1. The forest plot of the OS subgroups receiving ICI treatments or placebo plus chemotherapy.**

**Figure S2.** **Subgroup analysis of OS comparison between patients receiving PD1/PD-L1 plus chemotherapy and cadonilimab plus chemotherapy.**

**Abbreviations**: HR, hazard ratio; CI, confidence interval; Cad, Cadonilimab.

**Figure S3. The ranking of each treatment effect for OS of subgroups.**

P-score rankings are descriptive and should not be interpreted as definitive evidence of superiority.

**Figure S4. The forest plot and the ranking of effect for PFS.**

**Figure S4.** (A) The forest plot of the PFS of patients receiving ICI treatments or placebo plus chemotherapy (B) The forest plot comparing the hazard ratio of PFS of patients treated by anti-PD-(L)1 plus chemotherapy, cadonilimab plus chemotherapy and chemotherapy alone (reference group: anti-PD-(L)1 plus chemotherapy). (C) The ranking of each treatment effect for PFS. (D) The ranking of anti-PD-1/PD-L1 plus chemotherapy, cadonilimab plus chemotherapy and chemotherapy alone treatment for PFS. **P-score rankings are descriptive and should not be interpreted as definitive evidence of superiority.**

**Abbreviations**: HR, hazard ratio; CI, confidence interval; Cad, Cadonilimab; Che, chemotherapy.

**Figure S5. The forest plot of adverse events analysis.**

A

B

**Figure S5.** The forest plot of the grade 3-5 TRAEs (A) and SAEs (B) of patients receiving each ICI treatment or placebo plus chemotherapy in the fixed effect model (Anti-cadonilimab as reference).

**Abbreviations**: HR, hazard ratio; CI, confidence interval; Cad, Cadonilimab.

**Figure S6. The funnel plot of the analysis of efficacy and safety.**

**Figure S6.** (A and B) The funnel plot of the analysis of OS (A, Egger’s P=0.5685) and PFS (B, Egger’s P = 0.5666) benefits among different treatments. (C and D) The funnel plot of the analysis of Grade 3-5 TRAE (A, Egger’s P=0.7477) and SAE (B, Egger’s P = 0.1930) benefits among different treatments.

**Abbreviations**: Cad, Cadonilimab; Che, chemotherapy.

**Figure S7. Leave-one-out sensitivity analysis for the primary network meta-analysis of overall survival.**

**Figure S7. Leave-one-out sensitivity analysis for the primary network meta-analysis of overall survival.**

Panels A through G display the results of the primary network meta-analysis after sequentially excluding one study at a time. Each panel corresponds to the network with the indicated study omitted. Panel A: **ORIENT16 excluded**; Panel B: **RATIONALE305 excluded**; Panel C: **GEMSTONE-303 excluded**; Panel D: **CheckMate-649 excluded**; Panel E: **KEYNOTE-859 excluded**; Panel F: **KEYNOTE-062 excluded**; Panel G: **ATTRACTION-4 excluded**. The pooled hazard ratio (HR) and 95% confidence interval (CI) for the comparison of cadonilimab plus chemotherapy versus PD-(L)1 inhibitors plus chemotherapy are shown in each panel.

**Abbreviations**: HR, hazard ratio; CI, confidence interval; Cad, Cadonilimab.

**Figure S8. Leave-one-out sensitivity analysis for the primary network meta-analysis of progression free survival.**

**Figure S8. Leave-one-out sensitivity analysis for the primary network meta-analysis of progression free survival.**

Panels A through G display the results of the primary network meta-analysis after sequentially excluding one study at a time. Each panel corresponds to the network with the indicated study omitted. Panel A: **ORIENT16 excluded**; Panel B: **RATIONALE305 excluded**; Panel C: **GEMSTONE-303 excluded**; Panel D: **CheckMate-649 excluded**; Panel E: **KEYNOTE-859 excluded**; Panel F: **KEYNOTE-062 excluded**; Panel G: **ATTRACTION-4 excluded**. The pooled hazard ratio (HR) and 95% confidence interval (CI) for the comparison of cadonilimab plus chemotherapy versus PD-(L)1 inhibitors plus chemotherapy are shown in each panel.

**Abbreviations**: HR, hazard ratio; CI, confidence interval; Cad, Cadonilimab.
